# Neurosyphilis with ocular involvement and normal magnetic resonance imaging results affirmed by metagenomic next-generation sequencing

**DOI:** 10.3389/fcimb.2022.985373

**Published:** 2022-12-02

**Authors:** Xiaoli Zhou, Shengkun Peng, Tiange Song, Dandan Tie, Xiaoyan Tao, Li Jiang, Jie Zhang

**Affiliations:** ^1^ Department of Laboratory Medicine, Sichuan Provincial People’s Hospital, University of Electronic Science and Technology of China, Chengdu, China; ^2^ Sichuan Translational Medicine Research Hospital, Chinese Academy of Sciences, Chengdu, China; ^3^ Department of Radiology, Sichuan Provincial People’s Hospital, University of Electronic Science and Technology of China, Chengdu, China

**Keywords:** metagenomic next-generation sequencing (mNGS), *Treponema pallidum*, neurosyphilis, cerebrospinal fluid, normal magnetic resonance imaging, ocular involvement

## Abstract

The rapid and accurate identification of pathogenic agents is the key to guide clinicians on diagnosis and medication, especially for intractable diseases, such as neurosyphilis. It is extremely challenging for clinicians to diagnose neurosyphilis with no highly sensitive and specific test available. It is well known that the early transmission and immune evasion ability of *Treponema pallidum* have earned it the title of “stealth pathogen.” Neurosyphilis has complex clinical manifestations, including ocular involvement, which is infrequent and often overlooked, but its neuroimaging results may be normal. Therefore, it is important to find a new test that can detect the presence or absence of *Treponema pallidum* immediately for the diagnosis of neurosyphilis. We reviewed all the patients admitted to the Sichuan Provincial People’s Hospital between 2021 and 2022 who had ocular involvement and whose clinical samples were examined *via* metagenomic next-generation sequencing (mNGS), and we found 10 candidates for further analysis. The results of magnetic resonance imaging (MRI) were normal for four patients, and three of them met the diagnostic criteria for neurosyphilis confirmed by mNGS. In addition, the results of mNGS from the three patients were further validated using polymerase chain reaction (PCR). Five of the 10 patients had diplopia manifestations; two (20%) experienced abducens nerve palsies, two (20%) had eyelid drooping, and one (10%) had decreased vision. One of the 10 patients (10%) who was HIV positive and five patients had abnormal MRI results. To our knowledge, *Treponema pallidum* was detected by mNGS in patients with ocular involvement and normal MRI results for the first time. Given this situation, we recommend mNGS as a potential and supplementary tool for the diagnosis and differential diagnosis of neurosyphilis.

## Introduction

Syphilis has afflicted humans for more than 500 years, and this infection can progress to neurosyphilis, which is bothersome and serious. (Smibert et al., 2018). In a previous study, 40% of patients with syphilis developed neurosyphilis after *Treponema pallidum* invaded the central nervous system (CNS) (Ghanem et al., 2020), which is defined as neurosyphilis. In patients with neurosyphilis, the suppression of systemic immune responses may promote disease progression toward a neurological involvement, whereas CNS damage may be due to uncontrolled local host immune responses (Drago et al., 2016). Hence, neurosyphilis remains a relatively common complication that can occur at any stage of syphilis. Neurosyphilis can be easily treated with appropriate antibiotics, but it is difficult to diagnose because most patients are asymptomatic or have nonspecific symptoms. Neurosyphilis, if left untreated, can lead to syphilitic meningitis, meningovascular syphilis, general paralysis, tabes dorsalis, and ocular forms (Conde-Sendín et al., 2004). Neurosyphilis, which is caused by the infection of the nervous system by *Treponema pallidum*, is often overlooked because of its rarity. In addition, neurosyphilis with ocular involvement is also known as the “great masquerader” due to the multiple clinical features associated with this infection (Desideri et al., 2022).

*Treponema pallidum*, a helical to sinusoidal bacterium with outer and cytoplasmic membranes, is a species of spirochete in the Treponemataceae family. *Treponema pallidum* is a human obligate parasite, the genome of which is a circular chromosome of 1,138,006 base pairs (bp) containing 1,041 predicted coding sequences (open reading frames) (Fraser et al., 1998). *Treponema pallidum* can invade the central nervous system at the very early stage of infection. Moreover, *Treponema pallidum* establishes persistent infection by promoting the Treg response in the early stage of syphilis (Li et al., 2013). However, there are limitations to culture *Treponema pallidum* directly from the lesion exudate or tissue. Furthermore, diagnosing neurosyphilis is difficult because there is no highly specific and sensitive test yet available.

The clinical symptoms and imaging manifestations of neurosyphilis are diverse and lack specificity (Ropper and Longo, 2019). Its clinical manifestations include asymptomatic neurosyphilis, meningeal neurosyphilis, meningeal vascular neurosyphilis, paralytic dementia, tabes dorsalis, gumma swelling, ear syphilis, and syphilis-related eye diseases. Syphilis-related eye diseases can be seen at all stages of syphilis infection and can affect all structures of the eye; both eyes are often involved, which can be an isolated manifestation of neurosyphilis or a manifestation of tabes dorsalis or paralytic dementia, manifested as eyelid drooping, eye movement restriction, diplopia, decreased vision, blindness, conjunctival hyperemia, visual field defect, etc. Overall, neurosyphilis with ocular involvement needs to be distinguished among iridocyclitis, uveitis, conjunctivitis, scleritis, chorioretinitis, optic neuritis, optic nerve periarthritis, optic nerve retinitis, optic atrophy, oculomotor nerve palsy, abducens nerve palsy, pupil abnormalities, etc. Strikingly, patients with neurosyphilis may have no specific neuroimaging findings. Magnetic resonance imaging (MRI) examination remains an essential procedure for the detection of neurosyphilis although the results for two thirds of patients have been normal or nonspecific mild-to-moderate cerebral atrophy.

Currently, the diagnosis of neurosyphilis relies on the interpretation of serum and cerebrospinal fluid (CSF) serological tests as well as CSF characteristics, the patient’s exposure and treatment history, current symptoms, and neurological examination. Serological testing is the detection of antibodies and has been the main method for laboratory diagnosis of syphilis. Presumptive diagnosis of syphilis requires the use of two laboratory serological tests: non-treponemal (TRUST, VDRL, etc.) and treponemal testing (TPHA, TPPA, EIA, etc.) (Gonzalez et al., 2019). Non-treponemal tests detect a mixture of heterophile IgG and IgM although IgM does not help to stage syphilis accurately and should not be relied upon to determine the length of treatment. Above all, these tests require further optimizations and subsequent evaluations. The two types of serological tests (non-treponemal and treponemal) should be used together to reduce false-negative and false-positive rates (Workowski et al., 2021). Serological tests such as the newer automated treponemal tests (EIA, CIA, etc.) for syphilis are very sensitive in the early stages of infection (Janier et al., 2021). However, in the case of neurosyphilis, it is impossible to make a diagnosis by serological testing for syphilis alone, and the sensitivity and specificity of PCR are low, and as such, is not a recommended test (Marks et al., 2018). Additionally, the CSF test is imperfect and has no benchmark. In summary, the diagnosis of neurosyphilis is based on clinical evidence, abnormal results of treponemal and non-treponemal serologic assays, and CSF tests, which are time-consuming. In conclusion, direct detection of the pathogen *Treponema pallidum* from clinical specimens is of great importance for the early diagnosis of NS infection.

With the continuous updating and development of sequencing technology, next-generation sequencing (NGS) has gradually become an indispensable research method in the diagnosis of tumors, drug resistance, and infectious diseases (Zhang et al., 2020). Next-generation sequencing technologies include whole genome sequencing (WGS), transcriptome sequencing (RNAseq), whole exon sequencing (WES), metagenomic next-generation sequencing (mNGS), and etc. Since the first use of NGS to identify pathogens of infectious diseases in 2008, the widespread application of NGS in clinically difficult-to-diagnose diseases has kicked off (Chiu and Miller, 2019). Metagenomic next-generation sequencing (mNGS) has wide coverage and strong timeliness and is widely used in the accurate diagnosis of clinical infectious diseases. Furthermore, metagenomic next-generation sequencing (mNGS) technology can directly perform non-targeted, rapid, and accurate auxiliary diagnosis of pathogens in various diseases, such as respiratory system infections (Wilson et al., 2014), blood system infections (Li et al., 2018), and central nervous system infections (Yi et al., 2020). Overall, mNGS, as an emerging technology, can help in finding pathogens and providing evidence for diagnosis and treatment as soon as possible, which is not only beneficial in improving the prognosis of patients but also in the diagnosis and early treatment by clinicians.

## Methods

### Study design

This is an original study including a retrospective observational study and experimental validation of inpatients diagnosed with ocular involvement at our hospital between September 2021 and June 2022. Eligible patients were selected from the hospital information system, and their medical history, neurological examinations, peripheral blood and CSF laboratory testing, and neuroimaging examinations were recorded and evaluated. This research was approved by the Medical Professional Committee of the Sichuan Provincial People’s Hospital (Permit Number: 2022172) with a waiver for informed consent from the participants because of its retrospective nature.

### Study patients

We retrospectively reviewed the CT scans and MRI films of 10 inpatients with ocular involvement and whose clinical samples were examined by metagenomic next-generation sequencing during the period from September 2021 to June 2022. Clinical information and laboratory data were also retrieved from the files. The CT scans or MRI films were reexamined by one radiologist who was unaware of the patients’ clinical data, and these were retrospectively reviewed and interpreted for possible lesion.

### Clinical data

The data extraction included age, sex, symptoms, medical history, MRI results, CSF testing results, and blood testing results. The diagnostic criteria for NS were based on the 2021 sexually transmitted infections treatment guidelines of the U.S. Centers for Disease Control and Prevention and the 2020 European guideline on the management of syphilis. The details were as follows: (1) positive results for the treponemal (TPPA, EIA, TPHA, etc.) and nontreponemal tests (VDRL, RPR, TRUST, etc.); (2) white blood cell (WBC) counts of CSF > 5 cells/mm^3^; and (3) protein levels of CSF > 45 mg/dl.

### Sample collection

A lumbar puncture was performed to obtain cerebrospinal fluid according to standard procedures, and blood samples were obtained from the 10 patients with ocular involvement without clear origin in the Sichuan Provincial People’s Hospital from September 2021 to June 2022. White blood cell count, protein level, metagenomic next-generation sequencing (mNGS), and PCR validation were conducted on every CSF sample.

### Serologic tests

The syphilitic serologic tests for blood and CSF samples were performed using TRUST (WanTai, Beijing, China) and TPHA tests (Abbott, Chicago, USA) according to the manufacturer’s instructions. In general, TRUST tests were performed when TPHA tests were positive.

### Biochemical tests

Approximately 2 ml of CSF samples were collected in sterile tubes and analyzed within 1 h to determine the protein level by an Abbott ci16200 automatic clinical chemistry analyzer (Abbott, USA) and the white blood cell (WBC) count using an automatic blood cell BC-7500 analyzer (Mindray, Shenzhen, China).

### Cerebrospinal fluid sample processing and DNA extraction

CSF samples (600 μl each) from the patients were collected according to standard protocol. Lysozyme (7.2 μl) was added to the 600 μl of CSF sample in a 1.5-ml centrifuge tube and incubated in a metal bath at 30°C for the wall-breaking reaction. The mixture was then transferred to a new 2-ml centrifuge tube with 250-μl of 0.5-mm glass beads on a wall breaker and homogenized under the following conditions: 6 m/s, 45 s, two cycles, and an interval of 2 min. Then, 0.3 ml of the above mixture was transferred into a new 1.5-ml centrifuge tube for DNA extraction using the TIANamp Micro DNA Kit (DP316, TIANGEN BIOTECH, Beijing, China) according to the manufacturer’s protocol. The extracted DNA specimens were used for the construction of DNA libraries (Long et al., 2016).

### Construction of DNA libraries and sequencing

DNA libraries were constructed through DNA fragmentation, end repair, adapter ligation, and PCR amplification. Agilent 2100 (BGI Genomics Co.,Ltd., Shenzhen, China) was used for quality control of the DNA libraries. Qualified libraries were pooled, DNA nanoball (DNB) was made, and samples were sequenced by MGISEQ-2000 (BGI Genomics Co.,Ltd., Shenzhen, China) platform (Jeon et al., 2014).

### Bioinformatic analysis

The software fastp was used to trim and map the reads (Chen et al., 2018). High-quality sequencing data were generated by removing low-quality reads, followed by computational subtraction of human host sequences mapped to the human reference genome (hg19) using the Burrows–Wheeler Alignment (Li and Durbin, 2009), and removing low-complexity reads. The remaining data after the removal of low-complexity reads were classified by simultaneously aligning to the Pathogens Metagenomics Database (PMDB), consisting of bacteria, fungi, viruses, and parasites. The classification reference databases were downloaded from NCBI (ftp://ftp.ncbi.nlm.nih.gov/genomes/) containing 4,945 whole genome sequences of viral taxa, 6,350 bacterial genomes or scaffolds, 1,064 fungi related to human infection, and 234 parasites associated with human diseases.

### PCR of *Treponema pallidum* 47-kDa protein gene (TpN47)

PCR validation was performed with the primers referred by Orle et al. (1996) to amplify a fragment of the *T. pallidum* 47-kDa protein gene. The primers were as follows: KO3A 5′-GAAGTTTGTCCCAGTTGCGGTT-3′ and KO4 5′-CAGAGCCATCAGCCCTTTTCA-3′. DNA amplification was performed in a 20-μl of the reaction mixture containing primers, Rapid Taq Master Mix (Vazyme, Nanjing, China), and 4 μl of the DNA sample extracted from the clinical specimens (CSF and blood). PCR was performed in a thermal cycler (Thermo Fisher Scientific, Waltham, MA, USA) with the following parameters: 95°C for 5 min, followed by 40 cycles of denaturation (94°C), annealing (60°C), and extension (72°C) for 20 s at each step, and final extension at 72°C for 5 min. Amplified samples were stored at 4°C until analysis. The amplification products were analyzed by electrophoresis using 1.5% of agarose gel.

## Results

### Clinical data of the 10 patients with ocular involvement

**Figure 1:**
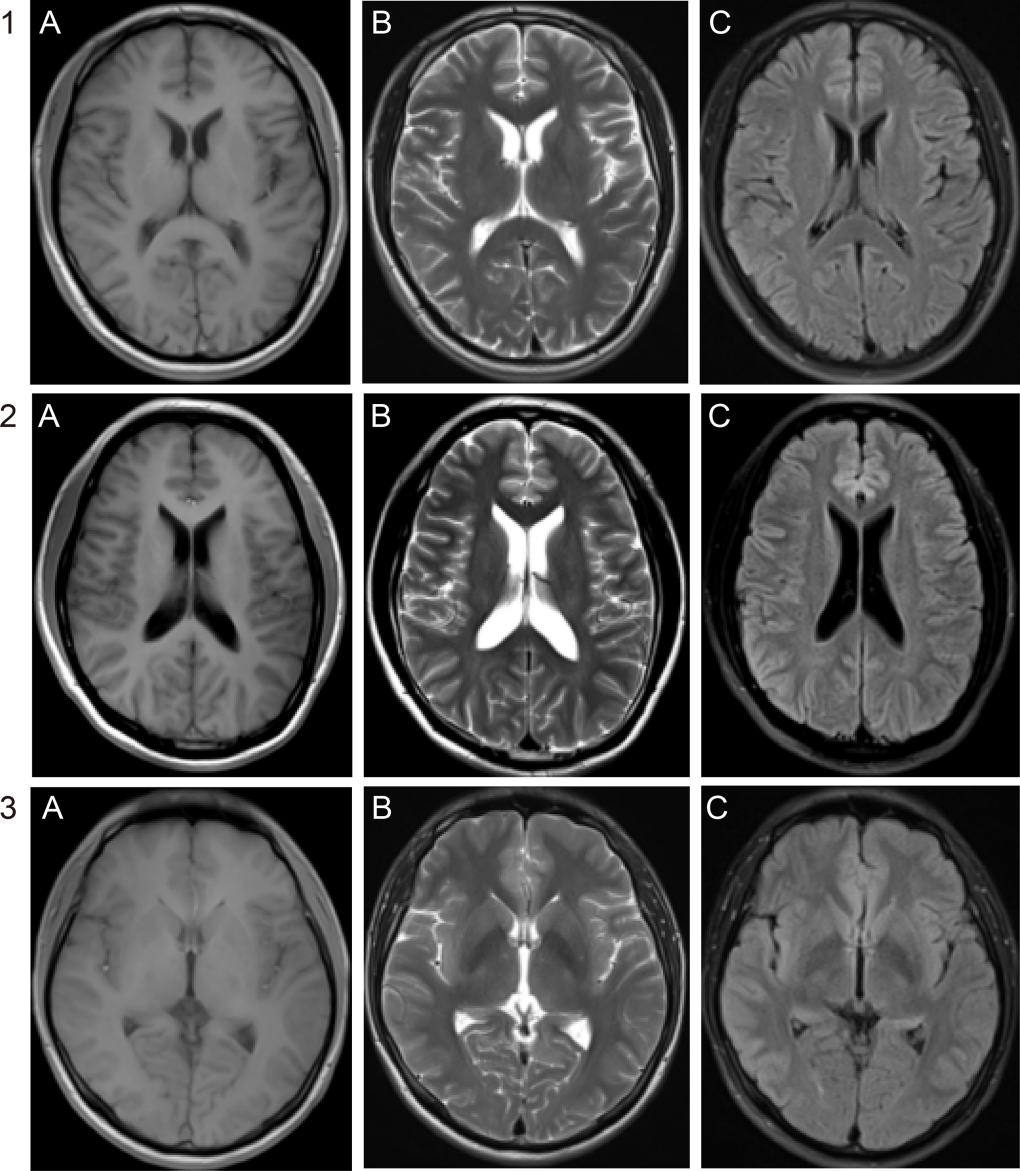
Brain MRI of the three patients. No abnormal signals were demonstrated on **(A)** T1WI, **(B)** T2WI, and **(C)** fluid-attenuated inversion recovery (FLAIR) of patients 1, 2, and 3.

Ten patients had ocular involvement including diplopia, abducens nerve palsies, eyelid drooping, and decreased vision (**Table 1**). One of the 10 patients had decreased vision manifestation, two (20%) experienced abducens nerve palsies, two (20%) had eyelid drooping, and five (50%) had diplopia. One of the 10 patients (10%) had a history of painless ulcers on the genitals, two had a history of syphilis and they said they had been cured, and their MRI results were all normal (**Figure 1**). Finally, the diagnosis of neurosyphilis was confirmed by mNGS for three patients with normal MRI results, which was also consistent with the serological and CSF results given in **Table 1**.

**Table 1:** Clinical characteristics and laboratory tests of the 10 patients with ocular involvement.

P	Age	Sex	Symptom	Syphilis history	Syphilis treatment status	MRI	Final diagnosis	Serological results			Cerebrospinal fluid results					
								TPHA	TRUST	PCR	TPHA	TRUST	White cells (/mm^3^)	Proteins (mg/dl)	mNGS	PCR
1	36	M	Diplopia	No	No	Normal	Neurosyphilis	+	1:32	Undetected	+	1:4	137 ↑	42	*Treponema pallidum*	Undetected
2	35	M	Diplopia	Yes, 5 years	cured	Normal	Neurosyphilis	+	1:128	Undetected	+	1:8	138 ↑	60 ↑	*Treponema pallidum*	Yes
3	32	M	Diplopia	Yes, 8 years	cured	Normal	Neurosyphilis	+	1:4	Undetected	+	–	13 ↑	18	*Treponema pallidum*	Undetected
4	68	M	Eyelid drooping	No	No	ND	Intracranial infection	–	ND	ND	ND	ND	740 ↑	1295 ↑	*Listeria ivanovii*	No
5	20	M	Decreased vision	No	No	Normal	Intracranial venous thrombosis	–	ND	ND	ND	ND	23 ↑	31	No	No
6	55	F	Abducens nerve palsies	No	No	Left abducens nerve tortuous vascular shadow	Infected cavernous sinus thrombosis	–	ND	ND	ND	ND	43 ↑	88 ↑	*Streptococcus constellatus*	No
7	56	F	Diplopia	No	No	Right otitis media	Cerebral ischemia	–	ND	ND	ND	ND	140 ↑	266 ↑	*Human gammaherpesvirus 4*	No
8	37	M	Diplopia	No	No	Left brainstem patch, streak shadow	Brainstem inflammation	–	ND	ND	ND	ND	51 ↑	27	*Human gammaherpesvirus 4*	No
9	56	F	Abducens nerve palsies	No	No	Bilateral basal ganglia, paraventricular ischemia	Skull basilar deformity	–	ND	ND	ND	ND	3	40	No	No
10	83	M	Eyelid drooping	No	No	Atrophy, right frontal infarction	Viral meningitis	–	ND	ND	ND	ND	109 ↑	145 ↑	No	No

↑ refers to the white blood cell (WBC) count of CSF >5 cells/mm^3^ or protein level of CSF >45 mg/dl. ND refers to ‘not done’.

### Laboratory findings

As shown in **Table 1**, seven patients had negative serological results, which can rule out the diagnosis of syphilis. The remaining three patients (30%, 3/10) had positive serum and CSF TPHA. In addition, the three patients also had positive serum TRUST (1 sero-TRUST titers ≤1:16 and 2 sero-TRUST titers ≥1:32). Two CSF samples (20%, 2/10) were TRUST-reactive with titers ≤1:16. Of the three patients, all had CSF pleocytosis; strikingly, only one had elevated CSF protein levels, which illustrated that CSF WBC count or protein level is also not the gold standard for the diagnosis of neurosyphilis.

### Next-generation sequencing confirms the diagnosis of neurosyphilis

**Figure 2:**
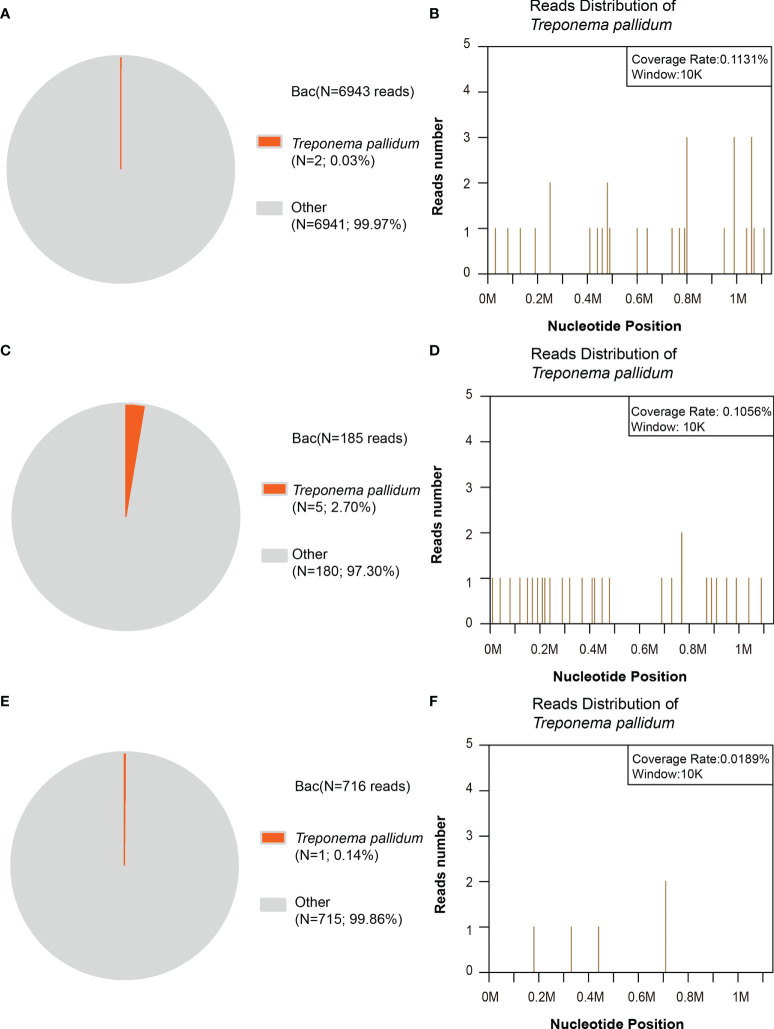
Next-generation sequencing (NGS) of the bacterial reads in the patients’ cerebrospinal fluid (CSF). **(A, B)** In patient no. 1, the distribution of bacterial reads (two of 6,943 reads; 0.03%) corresponded to *Treponema pallidum* with a coverage of 0.1131%. **(C, D)** In patient no. 2, the distribution of bacterial reads (five of 185 reads; 2.70%) corresponded to *Treponema pallidum* with a coverage of 0.1056%. **(E, F)** In patient no. 3, the distribution of bacterial reads (one of 716 reads; 0.14%) corresponded to *Treponema pallidum* with a coverage of 0.0189%. The others are commonly regarded as contaminating bacterial DNA from the environment and agents.

To draw a definitive pathogenic diagnosis and rule out infection by other pathogens, next-generation sequencing of CSF from the 10 patients with ocular involvement was also performed, and three of them were diagnosed with *Treponema pallidum* infection (**Figure 2**). *Treponema pallidum* DNA was detected in the CSF samples from the three patients whose MRI and CT results were negative. The number of unique reads of the identified *Treponema pallidum* gene ranged from 1 to 5 (0.03%–2.70%). Mapping of the detected reads to the reference *Treponema pallidum* genome resulted in coverage ranging from 0.0189% to 0.1131% with a depth of 1 and read lengths of 50, respectively. The number of unique reads, percentage, and coverage of the identified *Treponema pallidum* DNA sequences are shown in **Figure 2**. Interestingly, even though *Treponema pallidum* was at a relatively low pathogen level and the patients’ neuroimaging results were negative, the small number of pathogen reads was still detectable due to the massive sequencing data of mNGS to confirm the diagnosis.

### PCR validation

**Figure 3:**
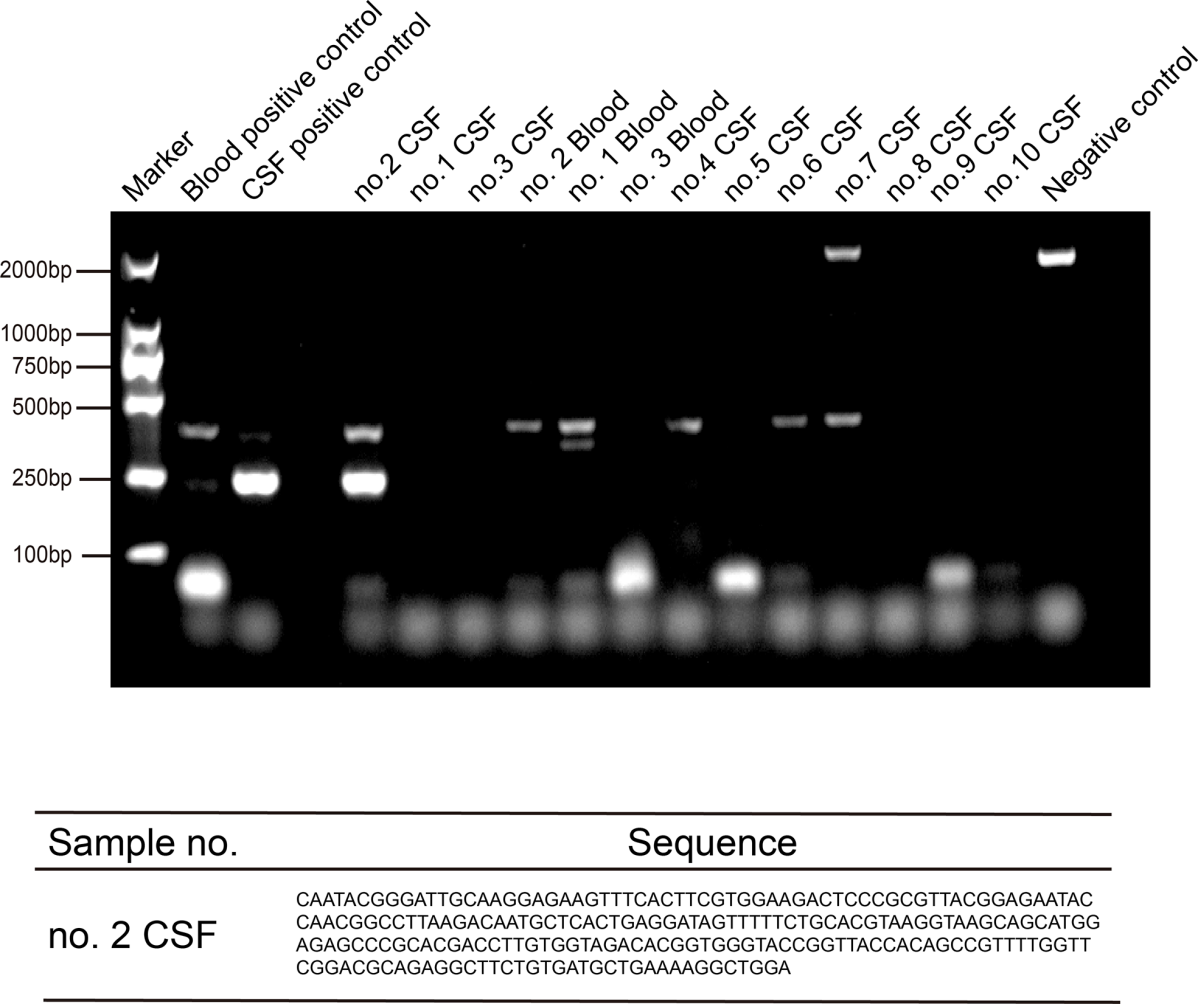
PCR and sequencing amplification of *Treponema pallidum* by TpN47 were followed by agarose gel electrophoresis to confirm *Treponema pallidum* sequences for three positive blood and 10 cerebrospinal fluid (CSF) samples. The blood positive control was from one patient’s blood sample (toluidine red unheated serum test [TRUST] titer was 1:256); the CSF positive control was from one patient’s CSF sample (positive serologic assays and metagenomic next-generation sequencing (mNGS); and the negative control was blank without sample.

We validated the NGS results using PCR analysis and Sanger sequencing. Specific primers for *T. pallidum* 47-kDa protein gene (260 bp) were designed (Palmer et al., 2003), and PCR was carried out for the 10 patients. To our surprise, only the second patient’s CSF PCR result was positive. The PCR results of the other two diagnosed patients were negative, possibly because the number of reads was small. The results showed that the amplification products (with DNA fragments of 250 bp) were consistent with our expectation, and the reads from Sanger sequencing matched the *Treponema pallidum* genome through NCBI Blast (**Figure 3**).

## Discussion

Neurosyphilis is highly treatable with appropriate antibiotics. Therefore, early recognition and management are essential. However, rapid diagnosis of neurosyphilis is challenging. Especially in immunocompetent individuals, neurosyphilis is more insidious with nonspecific symptoms, and diagnosis is difficult (Timmermans and Carr, 2004). Neurosyphilis with ocular involvement is infrequent, but the incidence of ocular pathology is high in patients coinfected with AIDS and syphilis, which might manifest as multiple fundus changes (Chen et al., 2021). All in all, on the one hand, the clinical manifestations of the disease are complex, and both MRI and CT results can be negative; on the other hand, *Treponema pallidum*, the pathogen of syphilis, is difficult to culture. Serological testing is the most common diagnostic method currently (Harris et al., 2022), and a negative serologic test can rule out neurosyphilis. However, no single test can be used to establish a diagnosis of neurosyphilis, an infection that can be easily missed or misdiagnosed (Du et al., 2021); a single specific deterministic testing remains lacking.

Neurosyphilis is caused by the pathogen *Treponema pallidum*, which can enter the CNS. With syphilis, there is a fierce battle between the host’s humoral and cellular immune responses, aimed at eliminating the infection, while *Treponema pallidum* manages to evade eradication and leads to chronic infection. Most patients can have an immune response to clear CNS invasion, and in a minority of patients who are immunocompromised or have immunodeficiency, syphilis may progress to asymptomatic or symptomatic NS. Neurosyphilis can be divided into early and late stages. The clinical manifestations in the early stage (e.g., meningitis, meningovascular syphilis, stroke, and acute altered mental status) usually appear within the first few months or years of infection. The presentation of the late stage (e.g., tabes dorsalis and general paresis) occurs 10 to >30 years after infection. Ocular or otic syphilis can occur at any stage with or without additional CNS involvement (Workowski et al., 2021).

The diagnosis of symptomatic neurosyphilis requires obtaining clinical features, conducting serological tests, and meeting the CSF criteria, whereas the diagnosis of asymptomatic neurosyphilis relies solely on the latter two (Workowski et al., 2021). However, serological tests for syphilis are relatively insensitive at the early stage of infection. Antibodies are undetectable by both non-treponemal and treponemal tests until infection progresses 1 to 3 weeks after chancroid formation (Larsen et al., 1995; Larsen et al., 1999). Therefrom, direct detection of *Treponema pallidum* from clinical samples has become a significant method for the early diagnosis of *Treponema pallidum* infection.

Moreover, neurosyphilis is classified into five types by imaging: asymptomatic neurosyphilis, meningeal syphilis (meningeal or scleromeningitis), syphilitic vasculitis, parenchymal syphilis (paralytic dementia), and gumma syphilis. The neuroimaging findings of neurosyphilis are usually cerebral infarction, leptomeningeal enhancement, or nonspecific white matter lesions (Tiwana and Ahmed, 2018). Neurosyphilis lacks specific imaging changes, which can also show similar imaging characteristics at different stages of the disease (Czarnowska-Cubała, 2015; Shang et al., 2020). In this study, the imaging findings of some patients were negative. Although the clinical examination was located in the nervous system, the imaging examination may not have meaningful findings.

Metagenomic next-generation sequencing (mNGS) enables non-targeted detection of nucleic acids from a range of potential pathogens, such as bacteria (Wilson et al., 2014), fungi (Christopeit et al., 2016), viruses (Piantadosi et al., 2018), and parasites (Li et al., 2021) present in clinical specimens. In the pathogenic diagnosis of CNS infectious diseases, cerebrospinal fluid mNGS technology has been gradually applied in clinical practice (Guan et al., 2016). Recently, Liu et al. (2022) reported that neurosyphilis with a high signal intensity on fluid-attenuated inversion recovery (FLAIR) was detected with 2,288 reads of *Treponema pallidum* by mNGS. In their study, they emphasize that more evidence from a large number of patients is needed to confirm mNGS as a supplementary method for the diagnosis and differential diagnosis of neurosyphilis. In this study, we were able to detect *Treponema pallidum* at a low pathogen level by mNGS, suggesting the sensitivity of the method. To our knowledge, this is the first time that *Treponema pallidum* was detected with a small number of reads by mNGS, in patients with ocular involvement and normal neuroimaging.

In this study, we retrospectively reviewed the CT scans and MRI films of 10 inpatients with ocular involvement and whose clinical samples were examined *via* metagenomic next-generation sequencing during the period from September 2021 to June 2022. Three out of the 10 patients had normal imaging results and were diagnosed with *Treponema pallidum* infection by mNGS. One read of *Treponema pallidum*, indicating the high sensitivity of mNGS, was also detected in patients with TRUST- or PCR-negative results, as we expected. Given this situation, we recommend NGS as a potential and supplementary tool for the diagnosis and differential diagnosis of neurosyphilis.

## Conclusions

Neurosyphilis is a complication of syphilis with potentially serious sequelae. *Treponema pallidum* invades the CNS, often occurring in the early stage of infection. Neurosyphilis with ocular involvement is uncommon in clinical practice and is often confused with eye disease. Moreover, the MRI and CT results may be normal. Therefore, early diagnosis of neurosyphilis with ocular involvement has important implications for patient prognosis. For the first time, we highlight the role of mNGS in diagnosing neurosyphilis with ocular involvement and normal MRI results even at a low pathogen level. This study confirms the significance of CSF mNGS as a diagnostic tool for CNS pathogen infections. Although not yet widely used, its use in CSF screening may provide the most reliable and timely clinical diagnosis of infectious diseases in the CNS in the near future.

## Data availability statement

The data analyzed in this study is subject to the following licenses/restrictions: All data supporting this study are reasonably available from the corresponding author. Requests to access these datasets should be directed to Jie Zhang, zhangjiespph@163.com.

## Ethics statement

The studies involving human participants were reviewed and approved by Medical Professional Committee of the Sichuan Provincial People’s Hospital (Permit Number: 2022172) with a waiver for informed consent from participants because of its retrospective nature.

## Author contributions

XZ, LJ, and JZ contributed to the conception and design of the study. XZ, TS, DT, and XT extracted the DNA and performed the NGS. XZ and JZ analyzed the data. JZ and SP helped to collect the clinical data and MRI images. XZ executed the PCR validation. XZ drafted the manuscript. JZ and LJ revised the manuscript. All authors contributed to the manuscript revision, and read and approved the final version of the manuscript.

## Funding

This work was supported by the National Natural Science Foundation of China (No. 81670893, No. 81702064).

## Acknowledgments

We sincerely thank the patients for participating in this original study.

## Conflict of interest

The authors declare that the research was conducted in the absence of any commercial or financial relationships that could be construed as a potential conflict of interest.

## Publisher’s note

All claims expressed in this article are solely those of the authors and do not necessarily represent those of their affiliated organizations, or those of the publisher, the editors and the reviewers. Any product that may be evaluated in this article, or claim that may be made by its manufacturer, is not guaranteed or endorsed by the publisher.

## Abbreviations

CSF, cerebrospinal fluid; mNGS, metagenomic next-generation sequencing; CNS, central nervous system; MRI, magnetic resonance imaging; CT, computed tomography; PCR, polymerase chain reaction; VDRL, venereal disease research laboratory; TPHA, *Treponema pallidum* hemagglutination assay; and TRUST, toluidine red unheated serum test.
